# The Impact of Irregular Menstruation on Health: A Review of the Literature

**DOI:** 10.7759/cureus.49146

**Published:** 2023-11-20

**Authors:** Ghalia M Attia, Ohood A Alharbi, Reema M Aljohani

**Affiliations:** 1 Medical Histology and Cell Biology, Mansoura University, Mansoura, EGY; 2 College of Medicine, Taibah University, Medina, SAU

**Keywords:** psychological and mental health, infertility, osteoporosis, anemia, quality of life, rheumatoid arthritis, cardiovascular diseases, type 2 diabetes mellitus, metabolism syndrome, irregular menstruation

## Abstract

Women are considered to have an irregular menstrual cycle if their cycle length is less than 21 days or more than 35 days, accompanied by less or very severe blood flow. The prevalence of menstrual cycle irregularities varies across countries. Irregular periods can occur due to changes in the body's levels of estrogen and progesterone hormones, which disrupt the normal pattern of the period. Menstrual irregularity has been found to be associated with various diseases and medical conditions, such as metabolic syndrome, coronary heart disease, type 2 diabetes mellitus, and rheumatoid arthritis. Anemia, osteoporosis, psychological problems, impaired quality of life, and infertility have also been recorded. Moreover, a significant correlation between irregular periods and the risk of developing pregnancy-related hypertensive disorders, as well as an increased risk of adverse obstetric and neonatal outcomes, has been proven. Therefore, irregular menstruation is considered an important health indicator among women. Physical, mental, social, psychological, and reproductive problems are often associated with menstrual irregularities. Thus, evaluating the factors associated with irregular menstruation is necessary to determine appropriate preventive and treatment strategies and to decrease the associated health problems. The aim of this review was to define normal and irregular menstruation, their types, and prevalence, to recognize the risk factors and causes of irregular menstruation, and to understand their impact on women's health.

## Introduction and background

Menstruation is a natural event and a unique experience for females of reproductive age [1]. The menstrual cycle is defined as the cyclic events that occur rhythmically during the reproductive period of a woman's life. It typically begins at the age of 12 to 15 years, marking the onset of puberty, and ceases at the age of 45 to 50 years, a phase known as menopause. The length of the menstrual cycle is generally 28 days, but under physiological conditions, it may vary between 20 and 40 days [2].

The prevalence of menstrual cycle irregularities among women ranges from 5% to 35.6%, depending on age, country of residence, and occupation [3].

Normal menstruation lasts 2 to 7 days and occurs every 21-35 days [4]. However, 14 to 25% of women experience irregular menstrual cycles, meaning their periods are heavier or lighter than usual, last longer than 35 days or shorter than 21 days, or they face other issues, such as abdominal cramps [5]. Menstrual irregularities also include bleeding or spotting between periods, bleeding or spotting after sex, menstrual cycle length varying by more than 7-9 days, and/or not having a period for 3-6 months [4,5].

Irregular periods can occur due to changes in the levels of the hormones progesterone and estrogen, which affect the normal pattern of the cycle. Common causes include polycystic ovary syndrome (PCOS), birth control pills, breastfeeding, excessive exercise, intrauterine devices, hyperthyroidism, or hypothyroidism [4]. Modifiable risk factors contributing to irregular menstruation include obesity, stress, and smoking [6].

Irregular menstruation can lead to different health consequences and is an indicator of women's health [7]. It has been linked to various illnesses and medical conditions, including metabolic syndrome [8], coronary heart disease [9], type 2 diabetes mellitus (DM) [10], and rheumatoid arthritis (RA) [11]. A strong link between the risk of developing pregnancy-related hypertensive disorders and irregular menstruation has also been demonstrated [12]. Menstrual irregularity before conception is associated with an increased risk of adverse obstetric and neonatal outcomes [13]. These health problems can affect work opportunities [4].

The rationale of the study is the commonality of irregular menstruation among women. Many women consider these irregularities normal, but in the long term, they may be associated with various medical conditions. This study highlights the most common impacts of irregular menstruation.

Search strategy

The current review was conducted using PubMed, Medline, Embase, and the Cochrane Library. Key terms such as "Normal Menstruation Cycle," "Irregular Menstruation," "Epidemiology and Risk Factors of Irregular Menstruation," and "Impact of Irregular Menstruation on Health and Development of Health Disorders" (including metabolic syndrome, type 2 diabetes mellitus, cardiovascular diseases, rheumatoid arthritis, quality of life, psychological and mental health, anemia, osteoporosis, fertility, and pregnancy problems) were used to search for related studies published within a specified period. We reviewed the abstracts of all related articles addressing the epidemiology of menstrual irregularities and the impact of irregular menstruation on health. The reference lists of included articles and recent reviews on the same topic were also examined.

Inclusion and exclusion criteria

All relevant articles published from 2013 to 2023 in the English language, with the purpose of addressing the epidemiology of menstrual irregularities and the impact of irregular menstruation on health, were included in our review. We excluded all reviews and duplicate publications to ensure the uniqueness and relevance of the data analyzed.

## Review

Normal menstruation cycle

The characteristics of the menstrual cycle are known by its length, duration, amount, and regularity [14]. Normal menstruation typically begins in teenagers between the ages of 11 and 14, lasts for 7 days or less, and follows a normal cycle length of 21 to 45 days with an average blood loss of 20-80 ml [15]. In the first two years after menarche, about half of the menstrual cycles are anovulatory due to the immaturity of the hypothalamic-pituitary-ovarian axis. Subsequently, the capacity for estrogen-positive feedback on the anterior pituitary improves, leading to the mid-cycle Luteinizing Hormone (LH) surge, ovulation, and the regulation of the menstrual cycle [16].

Changes in the ovary during each menstrual cycle occur in two phases: the follicular phase and the luteal phase, with ovulation occurring between these phases. Along with ovarian changes, uterine changes also occur simultaneously in three phases: the menstrual phase, proliferative phase, and secretory phase [2].

Irregular menstruation

Counseling women with irregular menstruation requires an understanding of menstrual cycle patterns [14]. Menstruation becomes irregular if the time between cycles changes, if more or less blood is lost during a period than normal, or if the duration of menstruation varies greatly from the norm [4]. The mean age of patients with menstrual irregularity was reported to be 33 years [17].

Rarely is a relevant pathology found to explain the irregularities. Nevertheless, irregularities in the menstrual cycle are common and can affect a woman during her reproductive years, raising concerns for her and her family. Physicians, patients, and parents should understand the normal pattern of the menstrual cycle to help assess the causes of irregular menstruation. This is important because some young girls seek medical advice for irregular menstruation that is actually within the normal range [18].

Menstrual irregularities are defined as follows: secondary amenorrhea if there is a cessation of menstruation for more than 6 months in the absence of pregnancy; oligomenorrhea if the cycle repeats about once every >35 days; polymenorrhea if the cycle repeats about once every <21 days (>5 pads/day fully soaked); hypomenorrhea if the duration of periods is <2 days with slight blood loss <1 pad/day; menorrhagia if the duration of periods is >8 days (>5 pads/day fully soaked); dysmenorrhea if menstruation is painful (spasmodic pain during the first 2 days of the cycle, lower abdominal pain radiating to legs) [17].

Epidemiology of irregular menstruation

The prevalence of irregular menstrual cycles ranges from 5% to 35.6%, varying by age, country of residence, and occupation [19]. This prevalence shows a wide range across countries: 29.7% in Saudi Arabia [20], 35.7% in India [21], 33.3% in Egypt [22], 64.2% in Nepal [23], 14.3% in Korea [24], and 5-15% in developing countries [25]. An Omani study revealed that dysmenorrhea is a common (94%) but undertreated menstrual disorder among Omani adolescent schoolgirls, with only 3% seeking medical attention despite severe pain [25]. In Dubai, 94.7% of adolescents reported dysmenorrhea as a significant issue [26]. A Saudi study found a 48.2% prevalence of menstrual irregularities during exams among female medical students, with dysmenorrhea (70.9%), lengthened cycles (45.6%), and heavy bleeding (41.9%) being most common [27].

Menstrual problems affect 75% of adolescent females and are the primary reason for gynecology visits [28]. Dysmenorrhea is the most frequent menstrual disorder leading to medical consultations for adolescents and their parents [29] and a major cause of long-term school absenteeism [28].

Studies on gynecological conditions are underrepresented in low- and middle-income Middle Eastern countries affected by war and political instability, such as Iraq, Syria, Libya, Sudan, Lebanon, Yemen, and Palestine, unlike in higher-income countries like the Gulf countries, Turkey, and Egypt, where women have greater access to healthcare and may be more thoroughly screened [30]. In these conflict-affected areas, young women may suffer from chronic pain and menstrual irregularities, but cultural norms may prevent them from seeking help, prolonging their suffering [30].

Risk factors of irregular menstruation

Environmental factors are likely responsible for determining menstrual flow, integrity, and regularity. Various environmental factors can affect menstrual cycle characteristics, including age, weight, physical activity, diet, caffeine consumption, smoking, exposure to organic solvents, occupation, workplace, medical conditions, and lifestyle factors [31]. Menarche occurring at age 15 or later has been related to experiencing long and irregular cycles [32]. Additionally, women with early menarche often tend to have irregular menstruation [25].

Causes of irregular menstruation

Doctors often cannot distinguish the reason for menstrual irregularities even after a thorough history taking and examination [17]. The cause of irregular menstruation differs according to age groups. Adolescents and perimenopausal females are more likely to experience an anovulation period, while the frequency of cancer and structural lesions rises with age [15,17].

The most significant cause of menstrual cycle irregularity is functional hypothalamic amenorrhea, associated with decreased gonadotropin-releasing hormone secretion and hypothalamic-pituitary-adrenal axis dysregulation [17]. Other causes include pregnancy, medications, ovarian and adrenal tumors, eating disorders, exercise-induced amenorrhea, and prolactinomas. Endocrine causes such as poorly controlled DM, Cushing's disease, PCOS, premature ovarian failure, thyroid dysfunction, late-onset congenital adrenal hyperplasia, and acquired conditions like stress-related hypothalamic dysfunction are also factors [6,33].

Research worldwide has investigated the prevalence of menstrual disorders and patterns across different ethnicities. Menstrual irregularities are heavily influenced by genetic predisposition [34], lifestyle patterns like dietary habits [35,36], physical activity [37], sleeping habits [38], and environmental exposures [39]. However, in developing countries like India, studying female reproductive health is challenging due to limited awareness and social stigma.

Obesity also raises the risk of developing menstrual dysfunction [40], though a variety of sex hormones play a role in these disorders as well. These diseases include a wide range of abnormalities such as menstrual cycle length, menstrual cycle length, irregular menstrual cycle, and menstrual blood loss. Obese women were found to have more irregular and longer menstrual cycles [41,42]. Furthermore, there is mounting evidence that menstrual cycle dysfunction in women is associated with breast cancer, endometrial cancer, CVD, and neurologic disorders [42,43]. Overweight women are at a higher risk of developing endometrial hyperplasia.

The underlying mechanism of our findings is unknown, but it could be explained by obesity being associated with high levels of estrogen [44], which is the main sex hormone influencing menstrual cycles, including blood loss. Women with extremely low body fat had significantly lower estrogen levels [45].

Impacts of irregular menstruation on women's health

Metabolic Syndrome

The key components of the metabolic syndrome have general consent, including impaired glucose tolerance, insulin resistance, type 2 DM, dyslipidemia, and hypertension (HTN) [46]. Compared to women with a history of normal menstrual cycles, women with irregular menstrual cycles tended to have a higher body mass index and a greater likelihood of reporting HTN, hypercholesterolemia, and DM [47].

A cross-sectional study among the general population showed that variables such as high triglyceride levels, high waist circumference, and low high-density lipoprotein cholesterol levels were significantly associated with irregular menstruation [8]. Irregular menstruation might be a marker of the increased risk of developing pre-DM and DM. Results may be explained by a high prevalence of PCOS that can lead to metabolic disorders in women with irregular menstruation, highlighting the importance of screening for metabolic disorders and giving recommendations promoting healthy lifestyles [47]. In a Brazilian study, PCOS, MS, and the criteria for MS were significantly more frequent in the subgroup with irregular menstruation [48].

Type 2 Diabetes Mellites

Impaired insulin secretion and sensitivity, dysfunction of beta cells, and impaired glucose tolerance are characteristics of type 2 DM [49]. Women with long or extremely irregular menstrual cycles have a significantly higher risk of developing type 2 DM [50]. Menstrual irregularity may be an indicator of underlying insulin resistance [51].

Amenorrhea and oligomenorrhea in young females can be symptoms of underlying metabolic disorders that significantly impact adult life. Generally, metabolic syndrome is known as a group of strongly related risk factors connected with insulin resistance/hyperinsulinemia, which predispose to CVD and influence their morbidity and mortality [52].

Cardiovascular Diseases

Women with a history of irregular cycles have an increased risk of coronary heart disease by 28% compared to women who have a regular cycle [53]. The likely reason is that irregular menstruation is related to PCOS, which has been associated with metabolic disorders and predisposes to CVD. In women with PCOS, many coronary risk factors have been well-defined, including high rates of obesity, glucose intolerance, increased insulin resistance, and HTN. Lipid disorders also include elevated levels of triglyceride, total cholesterol, low-density lipoprotein, and decreased levels of high-density lipoprotein [54]. Insulin resistance and obesity are both highly common in this population and are related to endothelial dysfunction and HTN [55]. Thus, vascular and metabolic changes characterizing the metabolic syndrome are closely related to the later development of CVD [56]. Women with irregular menstruation had a higher frequency of metabolic syndrome and PCOS [57].

Estrogen is a potent vasodilator due to nitric oxide production in healthy blood vessels. At the vascular level, estrogen mediates inflammation and oxidative stress, and over the long term, estrogen increases endothelial-cell growth and inhibits smooth muscle cell proliferation. Many lines of clinical evidence also relate hypoestrogenemia to premature CVD. One early clinical symptom of hypoestrogenemia is menstrual cycle irregularities [58].

Rheumatoid Arthritis

Most autoimmune disorders are more common in females. RA is a very common inflammatory rheumatic disease. Various approaches to study have been employed to investigate the effect of sex hormones on the development of RA. Current studies have concentrated on certain sex hormones and reproductive factors, such as androgens and estrogens. However, their contribution to the pathogenesis of RA is still unclear. Generally, the influence of sex hormones on the immune system and their interaction with genetic and environmental factors may possibly explain the increased prevalence of RA in females [59]. Women are 2-4 times more likely to develop RA than men, but conditions in women associated with excess estrogen and progesterone tend to be joint-protective. Women with RA show reduced joint symptoms during the postovulatory phase of the menstrual cycle and during pregnancy when the levels of estrogen and progesterone are high [60].

Very irregular menstrual cycles were correlated with a small increase in the risk of RA between the ages of 20 and 35 years [47]. Adrenal and gonadal androgens, which exert anti-inflammatory activities, are significantly decreased in inflamed tissues (that is, synovial fluid) during active RA in both male and female patients, supporting a proinflammatory milieu at least in RA joints [61]. The immune system is influenced by estrogens in both stimulatory and inhibitory ways. Estrogens at periovulatory to pregnancy levels support the survival of auto-reactive B- and T-cell clones and inhibit cell-mediated responses, such as the differentiation of Th17 cells. This includes the most potent 17 β-estradiol (E2), which binds to estrogen receptors, and its distribution varies across different tissues [59].

Quality of Life

It has been reported that 10% of women with dysmenorrhea suffer severely enough to render them incapacitated for 1-3 days each menstrual cycle, affecting their QOL, personal health, and having a global economic impact [28]. In addition, some girls are unaware that their bleeding patterns are abnormal, which can lead to significant long-term health consequences.

Menstruation is a normal aspect of being a woman and of gender identification. It is characterized as a monthly event and is a part of their life [19]. Considering that an irregular period is correlated with dissatisfaction and health-related anxiety, menstrual issues are considered important health measures for working women. Furthermore, irregular periods have a negative impact on efficiency at work [19]. Women often feel that their daily life is affected by their periods and menstrual symptoms. For example, they experience how their attention, endurance, and productivity are reduced at work or in school, making them feel less effective than they expected to be in social situations [1].

To investigate the quality of life connected to health, Ware created the SF-36 quality of life scale in 1987. This scale has two major titles, eight subdimensions, and 36 components. This scale uses positive scores, and an individual's quality of life increases if the score in each health area increases [35]. A cross-sectional study confirms that health-related quality of life in women with heavy menstrual bleeding in both physical and mental health is significantly reduced compared with women with normal menstrual blood loss (MBL), although the effect on mental health is greater than on physical health compared to the general population [62]. Moreover, heavy menstrual bleeding can have negative impacts on women's psychological condition, energy, efficiency at work, social relations, family life, and sexual functions [35].

Psychological and Mental Health

Menstrual cycle-dependent fluctuations in psychiatric symptoms can thus be considered common. Behavioral, psychological, and neuroendocrine influences are proposed as possible mechanisms underlying these fluctuations [63].

Irregular menstruation was associated with short sleep duration, depression, and other psychosocial health problems [64]. Menstrual cycle abnormalities were also related to eating disorders and depressive disorder symptoms [65, 66]. This may be because lower estrogen levels can disrupt general mental health, with increased rates of depression and anxiety. Another study revealed the association between menstrual irregularities and attention deficit hyperactivity disorder (ADHD) and psychiatric distress [66].

Young age at menarche and irregular menstruation influence mental health, especially in mood and anxiety symptoms. Therefore, during psychiatric evaluations, reproductive factors such as menarche age and irregular menstruation should be given attention [63].

Anemia

According to the World Health Organization, 29.9% of women aged 15-49 years suffered from anemia in 2019 [67]. Menstruation is the single most common cause of iron deficiency anemia in reproductive-age women [40]. The level of ferritin and physical functions decreased significantly as the duration of menstruation increased [18, 35]. Compromised iron stores undergo adaptive modifications, which eventually lead to limitations in hemoglobin production and iron deficiency anemia [68]. Heavy menstrual bleeding (HMB) is common in females and has been shown to reduce the levels of serum ferritin and hemoglobin used in anemia assessment and influence emotional and physical impairment. Heavy menstrual bleeding itself, and developing anemia and fatigue because of the bleeding, are recorded to decrease the QOL in females [35].

Osteoporosis

Amenorrhea is the cessation of menstrual cycles, characterized by low circulating estrogens. Hypoestrogenemia negatively affects calcium absorption through the intestine, leading to a reduction in calcium supply for bone absorption [58]. The effect of estrogen on bone tissue is significant. This impact is multifactorial and mostly associated with antiresorptive intervention. Recently, attention has been given to the role of estrogens in bone formation. Estrogens stimulate the synthesis of main growth factors such as transforming growth factor-beta (TGF-beta), bone morphogenetic protein 6 (BMP6), and insulin-like growth factor 1 (IGF1). Estrogens are also responsible for the increase of 1,25 (OH) D3, growth hormone, and progesterone expression receptors. Additionally, estrogen can have an impact on the suppression of RANKL (receptor activator of nuclear factor kappa B ligand) production. The RANK-RANKL system is a mediator in the formation of osteoclasts in response to identified stimuli and increases the expression of the osteoprotegerin gene. It also decreases proresorptive cytokine synthesis such as interleukin-1, interleukin-6, macrophage-colony stimulating factor, and tumor necrosis factor-alpha. In general, estrogen activates the bone remodeling units by stimulating bone formation and inhibiting bone resorption [69]. Therefore, long-term low estrogen in young females is related to osteopenia and osteoporosis.

Fertility

Reproductive activities, including transition from pregnancy, puerperium, and menopause, have a huge impact on the lives of women. Reproductive life is characterized and affected by the rhythm of the menstrual cycle and by its related hormonal changes. Any anomalies in this rhythm can affect several aspects of female life [6]. Getting irregular cycles has been linked to increased chances of subfertility. More than twice the odds of infertility were associated with irregular periods [70].

The primary factor in female infertility is failure to ovulate [71]. Irregular menstrual cycles may indicate ovulation problems. Anovulation may be due to a malfunction in the hypothalamus or pituitary gland, such as a result of a pituitary tumor, or if the hypothalamus does not secrete enough gonadotropin-releasing hormone to stimulate the release of FSH and LH, which leads to ovulation [72].

Pregnancy Problems

The role of the menstrual cycle is closely linked to fecundability, or the ability of a woman to become pregnant, and may affect the risk of her chronic disease [73]. Females who develop complications during pregnancy tend to have irregular menstrual cycles before conception [74]. Even mild cycle irregularities may indicate ovulatory disorders related to subfertility, and subfertile women who conceive spontaneously are considered to be at elevated risk of adverse obstetric and neonatal outcomes, including preeclampsia, antepartum hemorrhage, gestational HTN, preterm birth, low birth weight, and perinatal death [13].

Menstrual irregularity appears to be a separate risk factor for the potential development of Pregnancy-Induced Hypertensive Disorders (PRHDs). PRHDs are a relatively rare condition affecting nearly 2-5% of pregnancies, although they are responsible for significant maternal and fetal neonatal disease and death. One potential reason for this association is the high incidence of menstrual irregularities in PCOS patients [75]. Several authors have recently investigated the role of PRHDs in the risk of developing HTN and CVD later in life and concluded that there is an increased risk for both mothers and newborns [76].

## Conclusions

From the present review, it can be concluded that irregular menstrual cycles are a frequent source of concern for women. The menstrual cycle becomes irregular if the time between each period begins to change. This can occur as a result of changes in the levels of the hormones progesterone and estrogen, which affect the normal pattern of the cycle. The prevalence of irregular menstrual cycles had a wide range across countries. Menstrual irregularity was found to be associated with various diseases and medical conditions, such as metabolic syndrome, coronary heart disease, type 2 DM, and RA. Anemia, osteoporosis, psychological problems, affection of quality of life, and infertility were also recorded. Moreover, an important association between irregular periods and the risk of developing pregnancy-related hypertensive disorders and increased risk of adverse obstetric and neonatal outcomes was also proved. The early diagnosis and treatment of irregular menstruation may help decrease the infertility rate and the scale of other significant illnesses.
